# Cytokines in cerebrospinal fluid combined with machine learning improve the diagnostic accuracy and predict the progression of neurosyphilis

**DOI:** 10.3389/fimmu.2026.1677008

**Published:** 2026-04-20

**Authors:** Yu Zhao, Guohao Fan, Qi Sheng, Canghai Ma, Gaolei Dong, Qizheng Wang, Xiaoma Liu, Xi Xie, Jinhu Liang, Yun Peng, Shijun Wang, Yang Yang, Fuxiang Wang

**Affiliations:** 1 Shenzhen Third People’s Hospital, Second Hospital Affiliated to Southern University of Science and Technology, Shenzhen, China; 2 Shenzhen Clinical Research Center for Infectious Disease, Shenzhen, China

**Keywords:** cerebrospinal fluid, diagnostic, IL-2Rα, IP-10, machine learning, neurosyphilis

## Abstract

**Background:**

Syphilis is a sexually transmitted disease (STD) caused by *Treponema pallidum* (*TP*) which could progress to neurosyphilis affecting the nervous system. However, the pathogenesis of neurosyphilis remain unknown. In addition, current diagnosis of neurosyphilis is mainly based on serological tests and cerebrospinal fluid (CSF) analysis, with limitations in terms of sensitivity and specificity.

**Methods:**

From April 2021 to April 2023, 129 patients with syphilis were enrolled and divided into the non-neurosyphilis group (common syphilis, CS, n=19), asymptomatic neurosyphilis group (AN, n=77), symptomatic neurosyphilis group (NS, n=33) and a control group (n=15). Forty-eight cytokines/chemokines in the CSF samples were measured and analysed in combination with clinical indices. Feature selections were further analysed for the using different machine learning model for the diagnosis and progression predicting of neurosyphilis.

**Results:**

Machine learning-based feature selection identified a three-cytokine panel (RANTES, MIP-1β, and IL-3) that effectively distinguished syphilis patients from controls (AUC = 0.869, 95% CI: 0.821–0.917). For prediction of symptomatic neurosyphilis progression, IL-2Rα emerged as the optimal standalone biomarker, achieving an AUROC of 0.843 (95% CI: 0.784–0.902) for discriminating asymptomatic from symptomatic disease. Multivariable analysis confirmed IL-2Rα retained independent discriminatory power after adjusting for age (multivariable AUC = 0.876, 95% CI: 0.827–0.925). Age-stratified ROC analysis revealed IL-2Rα maintains robust diagnostic utility across all adult age groups, with optimal Youden-derived cut-offs of 21.82 U/mL (sensitivity 100.0%, specificity 85.7%, AUC 0.939) for 35–45 years; 25.48 U/mL (sensitivity 83.3%, specificity 88.9%, AUC 0.889) for 45–55 years; and 24.54 U/mL (sensitivity 73.7%, specificity 84.6%, AUC 0.789) for >55 years. The combination of IL-2Rα and IP-10 significantly improved predictive accuracy within specific age strata, achieving AUCs of 0.949 (35–45 years), 1.000 (45–55 years), and 0.814 (>55 years) when distinguishing symptomatic from asymptomatic neurosyphilis.

**Conclusion:**

In conclusion, IL-2Rα and IP-10 represent promising predictive biomarkers for neurosyphilis progression, with their combined application achieving high diagnostic accuracy for distinguishing symptomatic from asymptomatic disease. These findings establish a foundation for CSF cytokine-guided risk stratification in syphilis patients, although prospective validation in larger, age-matched cohorts is warranted to optimize clinical cut-offs and confirm generalizability across diverse populations.

## Introduction

Syphilis is an emerging sexually transmitted disease (STD) caused by *Treponema pallidum* (*TP*), which can cause non-neurosyphilis and neurosyphilis (1–3). Neurosyphilis is a clinical symptom caused by *TP* infection of the nervous system, which has occupied the fields of neurology and psychiatry for two centuries (4, 5). *TP* can invade the nervous system within days of initial infection, and subsequent neurosyphilis can be categorised as asymptomatic or symptomatic, which can occur at any stage of infection (6). Early stage of neurosyphilis is usually characterised by asymptomatic meningitis that manifested only by a cellular reaction in the cerebrospinal fluid (CSF), symptomatic neurosyphilis may also manifest itself in headaches, meningeal spasms, cranial nerve palsies, blindness or deafness (7). Late stages of the disease include generalised mild paralysis and spinal tuberculosis (8). The World Health Organization (WHO) estimates that 7.1 million adults aged 15 to 49 will be infected with syphilis in 2020 (9). In the United States, 176,713 cases of syphilis (all stages of syphilis and congenital syphilis) were reported in 2021, including 53,767 cases of the most contagious stage I and II syphilis with the incidence rising by 28.6% between 2020 and 2021 (10). In China, the incidence rate of syphilis was 33.08 per 100,000 in 2020, and increased to 34.05 per 100,000 in 2021 (11).

The laboratory diagnosis of neurosyphilis is based on abnormal results of serum and CSF serological tests and elevated CSF white blood cell counts and protein levels, but these tests are inadequate and without benchmarks (2, 5, 8). Almost all patients with neurosyphilis have positive serum syphilis spirochete tests during and after stage II syphilis, but in advanced neurosyphilis the titre declines over time especially after treatment (12). The CSF venereal disease research laboratory (VDRL) test is specific for neurosyphilis (except in cases of blood contamination), while the sensitivity is only 30 to 70% sensitive (13). The published data indicates that CSF *TP* PCR has a lower sensitivity for *TP* testing (ranged 40% to 70%) compared to CSF serological testing (12).

Inflammation is the body’s primary coordinated defence against tissue damage caused by injury or infection and involves activation of both innate and acquired immune responses. However, an active immune response following infection, also known as a cytokine storm, has been found to be associated with excessive levels of pro-inflammatory cytokines and widespread tissue damage (14). The cellular immune response plays an essential role in the clearance of *TP* infection, which may cause a strong host-specific immune response and large amounts of cytokines (15). However, the host excess inflammatory and immune response contributes to syphilis-related symptoms and tissue damage (7, 16, 17). Thus, the anti-inflammatory immune response appears to play an important role in balancing excessive pro-inflammatory responses (17). In recent years, the detection of cytokines in CSF has also been used to diagnose neurosyphilis. mokine ligand (CXCL 13, CXCL8, CXCL10) and macrophage movement inhibitory factor have also proved to be valuable biomarkers for differentiating neurosyphilis from non-syphilitic/syphilis in HIV-negative patients (18–20). Positive correlation between CSF myeloid cells 2 (the triggering receptor expressed on myeloid cells-2, TREM2) and CSF neurofilament light proteins suggests a link between microglia activation and neuronal damage in neurosyphilis (21). However, there are some limitations in previous studies on this area, such as limited number of cases and fewer cytokines tested.

Machine learning (ML) has demonstrated transformative potential in medical diagnostics. By leveraging algorithms that detect complex patterns within high-dimensional, multimodal data, including clinical variables, imaging, genomics, and proteomics (22), ML has significantly improved diagnostic accuracy, efficiency, and the ability to detect diseases early (23). For example, in the case of cardiovascular disease, models such as random forests have made it possible to predict coronary artery disease non-invasively and with high accuracy (24, 25). In oncology, support vector machines and deep neural networks are routinely used to classify and assess the prognosis of conditions such as breast cancer and leukaemia (24, 26). Similarly, ML approaches that integrate biomarkers with immune profiles have successfully stratified patients with neurodegenerative disorders (e.g., Alzheimer’s disease) (27) and infectious diseases (e.g., SARS-CoV-2 infection) according to disease status (28). Notably, ML frameworks based on cytokine profiling have shown strong discriminatory performance in diagnosing various inflammatory and infectious encephalopathies (27, 28). Together, these advances suggest that applying ML to high-throughput cytokine data could overcome the limitations of conventional diagnostic strategies and provide a new way to identify syphilis and its neurological complication, neurosyphilis.

In this study, we systematically characterized the comparative cytokine/chemokine expression profiles of syphilis with different disease severity, and identified biomarkers for progression and diagnosis of syphilis and symptomatic neurosyphilis using ML.

## Methods

### Patient information and data collection

In this prospective study, a total of 129 patients with syphilis admitted to Shenzhen Third People’s Hospital during April 2021 to April 2023 were enrolled, and 15 cases with neurological symptoms while negative for *TP* and other common pathogens for nerve system infection were included as the control patients (**Supplementary Table 1**). Inclusion criteria for controls included having a clinical indication for cerebrospinal fluid (CSF) analysis due to neurological symptoms, negative treponemal (TPPA/TPHA) and non-treponemal (RPR/VDRL) serological tests, negative CSF polymerase chain reaction (PCR) results for (Herpes simplex virus (HSV)-1, HSV-2, Varicella-zoster virus, Cytomegalovirus, Epstein-Barr Virus, Human herpesvirus (HHV)-6, HHV-7, Parechovirus, Enterovirus, Mumps virus, Measle virus, Haemophilus influenzae, Streptococcus pneumoniae, Staphylococcus aureus, Listeria monocytogenes, Neisseria meningitidis, Streptococcus agalactiae, Cryptococcus neoformans, Escherichia coli, Dengue virus, Japanese encephalitis virus, West Nile virus and Tick-borne encephalitis virus. And no evidence of autoimmune encephalitis (negative neural autoantibodies) or other inflammatory central nervous system (CNS) disorders on magnetic resonance imaging (MRI) or clinical follow-up. Exclusion criteria apply to all groups, including: prior use of antibiotics, antiviral medications, or corticosteroids before cerebrospinal fluid collection, as well as incomplete demographic, clinical, or cytokine data. The 129 patients with syphilis were further divided into three groups based on the disease severity, with 19, 77 and 33 patients belonging to the non-neurosyphilis (common syphilis, CS), asymptomatic neurosyphilis (AN) and symptomatic neurosyphilis (NS), respectively(**Supplementary Figure 1**). For the NS group, the patients were diagnosed by specialists based on clinical symptoms following the professional standards (Diagnosis for syphilis, WS 273-2018).

This study was conducted in accordance with the ethical standards of the Declaration of Helsinki and was approved by the Ethics Committee of Shenzhen Third People’s Hospital, Shenzhen, Guangdong Province, China (Approval No (2023–066–03).; Approval Date: [16 April 2021]). Written informed consent was obtained from all participants prior to their inclusion in the study. In cases where patients lacked the capacity to consent due to severe neurological symptoms (e.g., altered mental status), written informed consent was obtained from their legally authorized family members or guardians.

### Measurement of CSF cytokines

The CSF samples from laboratory-confirmed syphilis patients and controls were collected via lumbar puncture prior to the initiation of any antimicrobial or immunomodulatory therapy, during the patient’s initial hospital admission. Samples were processed within 30 minutes of collection: centrifuged at 2,000 × g for 10 minutes at 4 °C, aliquoted, and stored at –80 °C until cytokine analysis. Concentrations of 48 cytokines/chemokines were measured in duplicate using the Bio-Rad Bio-Plex Pro Human Cytokine 48-Plex Screening Panel (Bio-Rad, Hercules, CA, USA) on a Luminex 200 system, following manufacturer protocols (14, 29).

Standard curves were generated from provided standards (8-point serial dilutions), and sample concentrations calculated using Bio-Plex Manager software (v6.1). Values below the lower limit of quantification (LLOQ) were assigned half the LLOQ value. Intra-assay CV was <10% and inter-assay CV <15%. No samples showed values above the upper limit of quantification. Measurements were performed by technicians blinded to clinical group assignment.

### Statistical analysis

The chi-square test and analysis of variance (ANOVA) were used to compare whether the CSF cytokine levels were statistically significant in the control, CS, AN and NS groups. Principal Coordinates Analysis (PCoA) was performed based on a Euclidean distance matrix derived from the concentrations of 48 cytokines to visualize the overall differences in cytokine profiles among different patient groups. We employed a two-stage machine learning approach. First, LASSO logistic regression was used for high-dimensional feature selection to identify the most discriminative cytokines from the 48 measured. LASSO logistic regression was performed using the glmnet package (v4.1-7) in R. The optimal regularization parameter (λ) was determined via 10-fold cross-validation with 100 repetitions, selecting λ.1se (the largest λ within one standard error of the minimum) to balance model parsimony and predictive accuracy. The data were randomly split into training (70%) and validation (30%) sets with stratification by disease status to maintain group proportions.

Subsequently, the diagnostic performance of the selected cytokine panel was evaluated using multiple classifiers—including logistic regression, support vector machine (SVM), random forest, and XGBoost—with model performance compared via receiver operating characteristic (ROC) curve analysis. Multiple machine learning classifiers were implemented using scikit-learn (v1.3.0) and XGBoost (v2.0.0) in Python. Models included: (1) Logistic Regression with L2 regularization (C = 1.0); (2) Random Forest (n_estimators=500, max_depth=10, min_samples_split=5); (3) Support Vector Machine with RBF kernel (C = 1.0, gamma=‘scale’); and (4) XGBoost (max_depth=6, learning_rate=0.1, n_estimators=100). Hyperparameters were optimized using 5-fold cross-validated grid search. All continuous variables were standardized (z-score normalization) prior to model training. Model performance was evaluated using stratified 10-fold cross-validation repeated 10 times to ensure robustness. The ROC for CSF cytokine levels was estimated separately for patients in the control, CS and AN groups compared to the NS group.

To address age as a potential confounder and establish clinically applicable thresholds, we performed age-stratified ROC analysis and stratified analyses across three age groups based on sample distribution: 35–45 years, 45–55 years, and >55 years. For each stratum, separate logistic regression models were constructed to calculate AUC values. Optimal cut-off values were determined by maximizing the Youden index (J = Sensitivity + Specificity − 1). This approach allowed us to assess whether IL-2Rα maintains diagnostic independence from chronological age and to provide age-adjusted clinical decision thresholds. All statistical tests and figures were performed with SPSS 26.0, R and Python. P values of 0.01 - 0.05, 0.001 - 0.01 and <0.001 were considered statistically significant, highly significant and extremely significant, respectively.

## Results

### Epidemiological and clinical characteristics of the cohort

Among the 129 patients with syphilis, 66 patients (51.2%) were male, with no statistical differences among the three groups in terms of gender and BMI (*P=*0.081 and *P=*0.886) (**Supplementary Figures S2A, D**). The mean age of patients with non-neurosyphilis and asymptomatic neurosyphilis was 41.8 ± 3.0 and 38.3 ± 1.5 years respectively, mainly concentrated between 20–60 years (96.8% and 89.6% respectively) (**Supplementary Figure 2B**). Patients with symptomatic neurosyphilis patients were significantly older, with mean ages of 57 ± 2.3 years and most individuals (97%) older than 40 years (**Supplementary Figure 2C**). Given the significant age disparity between AN and NS groups (mean difference: 19 years), which could confound cytokine-level interpretations due to age-related immune activation, we performed age-stratified analyses to determine whether IL-2Rα retains independent diagnostic utility. No statistical differences were found in the duration of infection history, CSF and blood white blood cell count (WBC), comorbid underlying diseases (**Supplementary Table 1**). Clinical symptoms of CS mainly included neurological abnormalities (23 cases, 69.7%) including babbling, hallucinations, memory loss, irritability, and emotional instability, and muscular disorders (10 cases, 30.3%) manifested as muscle pain, unsteady walking, facial numbness, limb weakness, etc.; visual abnormalities (5 cases, accounting for 15.2%); and skin rash (3 cases, accounting for 9.1%). Except for the symptoms of headache and rash, which were not statistically different among the three groups (*P=0.322 and P = 0.123*), all other symptoms were statistically different (**Supplementary Table 1**).

### Comparison of cytokines among the Control, CS, AN and NS group

**Figure 1:**
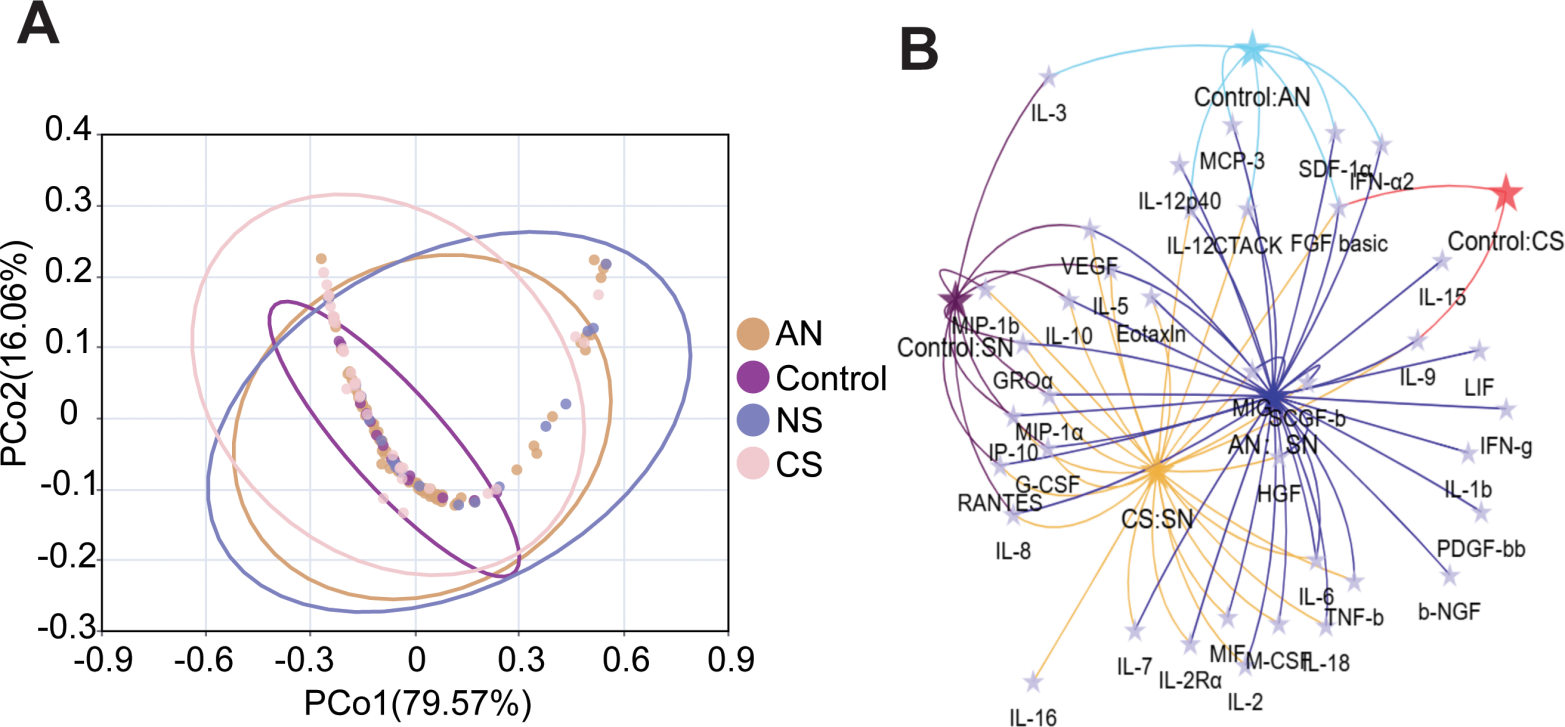
Comparison of cytokines among the Control, CS, AN and NS in CSF samples. **(A)** Principal component analysis (PCA) of cytokine among Control, CS, AN and NS in CSF samples. Each plot represents one sample. **(B)** Venn diagram illustrating the differentially expressed cytokines for each group, both specific and commonly shared cytokines.

A panel of 48 cytokines was measured in 139 CSF samples and the data were preprocessed for further analysis. Cytokine expression was first characterized across the four groups. Principal co-ordinates analysis (PCoA) showed that PCoA1 and PCoA2 explained 79.57% and 16.06% of the percentage variance, respectively (**Figure 1A**). To further directly compare cytokine differences, pairwise comparisons were conducted among the four groups. Of the 48 cytokines, 18 showed significant differences between at least one control group. 2 cytokines were significantly different between Control and CS groups, 7 cytokines demonstrated significant differences between Control and AN group, and 12 cytokines exhibited significant differences between Control and NS groups. No significant cytokine differences were observed between the CS and AN groups, 27 cytokines were significantly different between CS and NS groups, and 36 cytokines were significantly different between the AN and NS groups (**Figure 1B**; **Supplementary Figure 3**). Notably, 10 cytokines (G-CSF, IL-5, IL-8, IL-10, IP-10, MIP-1a, RANTES, VEGF, GROa, and IL-2Rα) were significantly elevated in the NS group compared to all other groups (**Supplementary Figure 3**), suggesting a distinct inflammatory signature in symptomatic neurosyphilis (**Supplementary Figure 3**). Whereas there were cytokines with at least one significant difference between the control group and the other groups, no cytokines with a common significant difference among the three were found, so multiple cytokines combinations may be needed to diagnose patients with syphilis in CSF.

**Figure 2:**
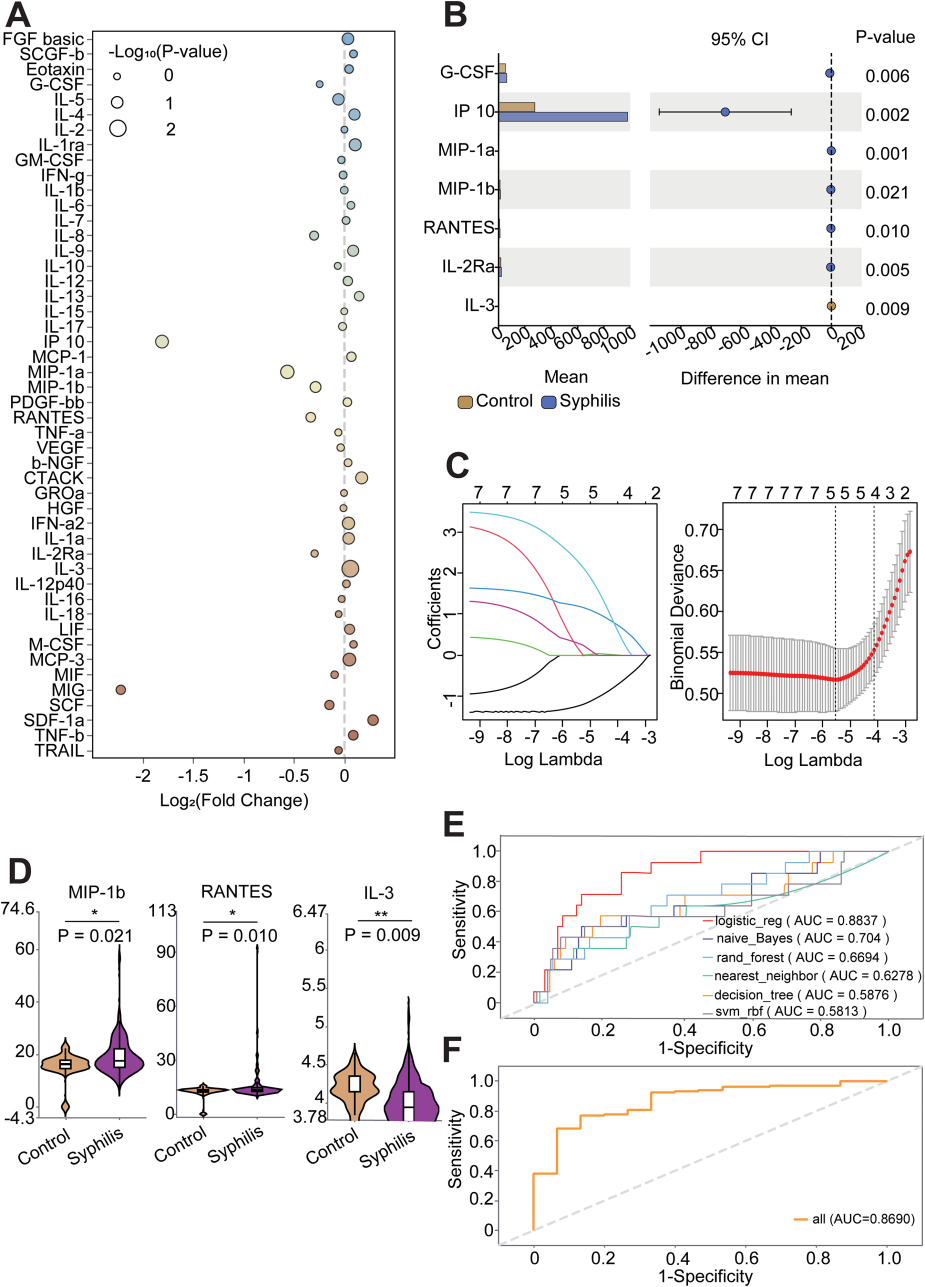
Comparison of cytokines among the Control and Syphilis group (CS, AN and NS) in CSF samples. **(A)** Graph of difference bubbles illustrated the differential expression of 48 cytokines in Control and syphilis patient groups. **(B)** Subgroup difference plots showed the characteristics of the 7 cytokines initially screened with significant differences in the Control and Syphilis groups. **(C)** Lasso regression was further analysed for 7 differentially expressed cytokines among the Control and Syphilis groups. **(D)** Violin plot displaying the 3 differentially expressed cytokines in **(B)**. *p < 0.05, **p < 0.01, ***p < 0.001. **(E)** Machine Learning Models Further demonstrate diagnostic and predictive capabilities of 3 cytokines with significant differences. **(F)** Traditional ROC curves characterise the detection efficiency of three cytokines.

To find biomarkers for the diagnosis of syphilis, we comparatively analysed the expression of 48 cytokines in Control and Syphilis groups (CS, AN and NS), which showed that 8 out of 48 cytokines Granulocyte colony stimulating factor (G-CSF) (log_10_P-value=2.1887), interferon γinducible protein-10 (IP-10) (log_10_P-value=2.7421), Macrophage inflammatory protein-1 alpha(MIP-1a) (log_10_P-value=3.0976), Macrophage inflammatory protein-1 beta (MIP-1b) (log_10_P-value=1.6685), regulated upon activation normal T cell expressed and secreted (RANTES) (log_10_P-value=1.9879), Interleukin-2 Receptor SubunitAlpha (IL-2Rα) (log_10_P-value=2.2837),Interleukin-3(IL-3) (log_10_P-value=2.0508) and monokine induced by IFN-γ(MIG) (log_10_P-value=1.4276) differences were statistically significant (*P* < 0.05) (**Figure 2A**). Among these, seven cytokines showed robust differential expression (MIG was excluded due to small differences) (**Figure 2B**). The 7 cytokines screened were further analysed using LASSO regression, and 3 cytokines were screened to be optimal for predicting Control and Syphilis (Lambda=0.0047), RANTES (0.0864), MIP-1b (+0.0481) and IL-3 (-0.0697) (**Figures 2C, D**). We validated the diagnostic and predictive effects of the three selected cytokines using various machine learning models, and the logistic regression analysis model had the best predictive effect (AUC = 0.8837) (**Figure 2E**), and in order to validate this effect, we characterised the predictive effect of the three cytokines using the traditional ROC curve, and the results showed that the AUC = 0.869, also with a strong diagnostic effect (**Figure 2F**). In contrast, RANTES, MIP-1b and IL-3 individual cytokines or any two-cytokine combination showed lower diagnostic performance than the full three-marker panel (**Supplementary Figure 4**).

### Differential expression profile of cytokines in syphilis patients with different disease severity

PCoA of cytokine profiles in CS versus neurosyphilis groups, which showed that PCoA1 and PCoA2 explained 79.81% and 15.80% of the percentage variance, respectively (**Supplementary Figure 5A**). In order to find biomarkers that induce neurosyphilis, we comparatively analysed the expression of 48 cytokines in CS group and Neurosyphilis (AN and NS), and the results showed that 8 out of 8 cytokines G-CSF (log_10_P-value=2.469), IL-6 (log_10_P-value=1.695), IL-10 (log_10_P-value=1.783), IP-10 (log_10_P-value=2.545), MIP-1a (log_10_P-value=3.605), Tumour Necrosis Factor Alpha(TNF-1a) (log_10_P-value=1.903), IL-2Rα (log_10_P-value= 2.631), and MIG log_10_P-value=1.382) with statistically significant differences (P<0.05) (**Supplementary Figure 5B**). Notably, these cytokines all exhibited upregulated expression. We then further analysed the screened 8 cytokines using LASSO regression to screen out the only cytokine MIP-1a (**Supplementary Figures 5C**, **3D**). Validation of the machine learning model for diagnostic and predictive efficacy showed that the logistic regression analysis model predicted AUC = 0.7124 (**Supplementary Figure 5E**), and the predictive efficacy of MIP-1a was characterised using a conventional ROC curve, which showed AUC = 0.636, The results demonstrated that MIP-1a could not effectively predict the disease progression from non-neurosyphilis to neurosyphilis. This may be related to the lack of significant differences in CSF cytokines between patients with asymptomatic neurosyphilis and those with non-neurosyphilis.

### IL-2Rα is strongly correlated with the progression of symptomatic neurosyphilis

**Figure 3:**
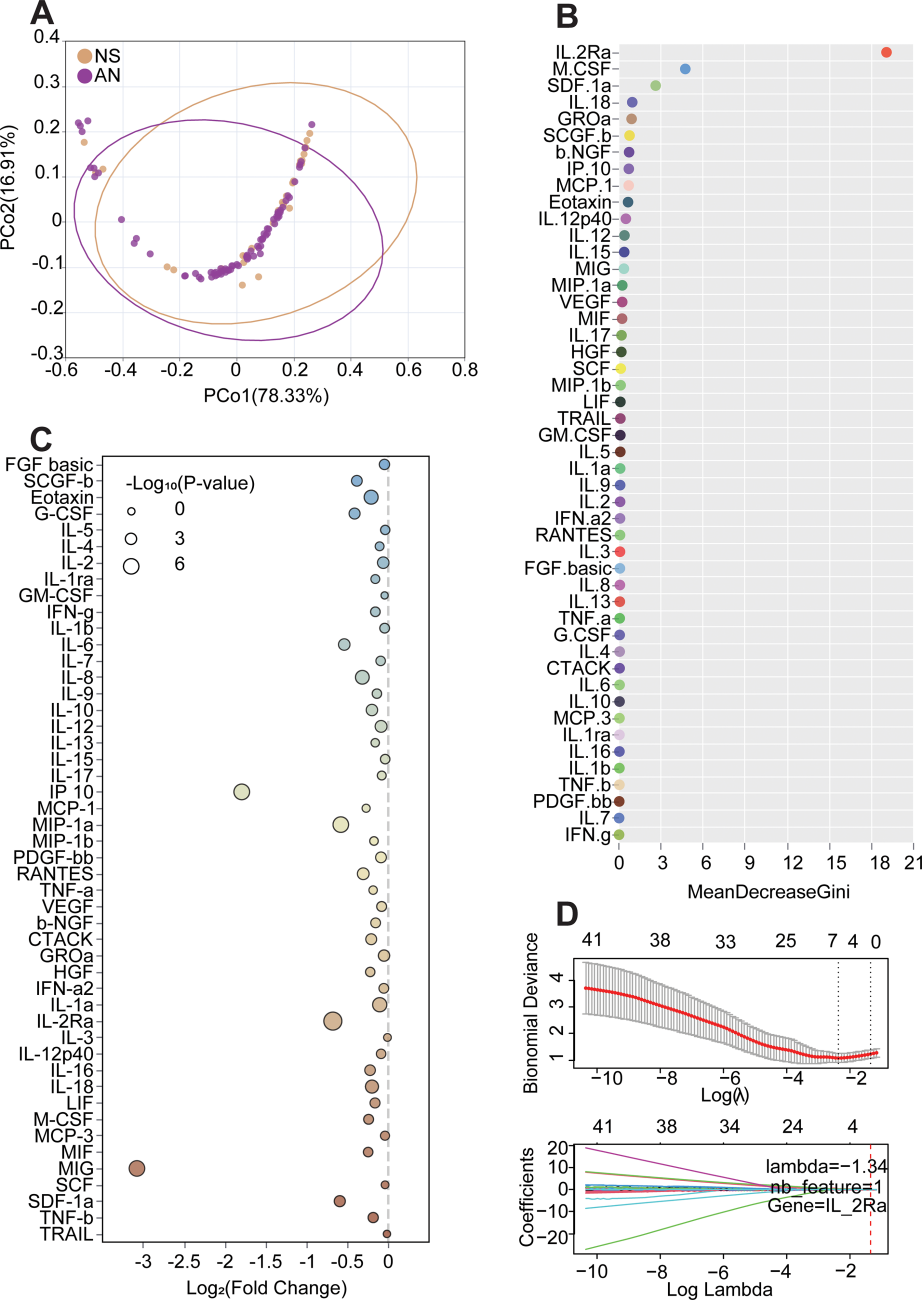
Comparison of cytokines among the AN and NS in CSF samples. **(A)** PCoA of cytokines among AN and NS in CSF samples. Each plot represents one sample. **(B)** Random forest model(RF) of 48 cytokines among CS AN and NS in CSF samples. **(C)** Graph of difference bubbles illustrated the differential expression of 48 cytokines in AN and NS patient groups. **(D)** Lasso regression was further analysed for 48 differentially expressed cytokines among the Control and Syphilis groups.

To demonstrate that the excessive expression of potential key cytokines may trigger the clinical symptoms of neurosyphilis. To identify drivers of symptomatic progression, cytokine differences between AN and NS were analysed. First, we performed PCoA on 48 cytokines in the AN and NS groups. The results demonstrated that PCoA1 and PCoA2 accounted for 78.33% and 16.91% of the variance, respectively (**Figure 3A**). To further compare the expression of the 48 cytokines between AN and NS, we employed random forest (RF) modelling to identify and validate key cytokines affecting neurosyphilis. The results suggested that IL-2Rα could be a crucial cytokine influencing the progression of neurosyphilis (MeanDecreaseGini=17,75) (**Figure 3B**). Differential bubble plot analysis identified 36 differentially expressed cytokines, all of which were up-regulated, indicating a potential cytokine storm leading to neurological damage and clinical symptoms of neurosyphilis. Among these, IL-2Rα (log_10_P-value=2.119) was particularly significant (**Figure 3C**). To further screen and validate important cytokines, we used LASSO regression analysis(Lambda=-1.34), which confirmed IL-2Rα (-0.0156) as the sole selected cytokine, consistent with the random forest model (**Figure 3D**). IP-10 was the next most significant cytokine, also demonstrating strong predictive ability for disease progression. Therefore, further analysis using the normalised confusion matrix revealed that the predicted label and True label consistency was 0.88 for AN and 0.64 for NS. IL-2Rα was superior for predicting AN (**Supplementary Figure 6 C**). After we added the cytokine IP-10, the results showed that the predicted label and True label of NS increased significantly to 0.82. IP-10 combined with IL-2Rα improved the value of predicting NS (**Supplementary Figure 6D**). Consequently, we considered IP-10 as an auxiliary factor to IL-2Rα and constructed ROC curves using various machine learning models for IL-2Rα alone and IL-2Rα combined with IP-10. The results indicated that the logistic regression model exhibited the best predictive performance (AUC = 0.941 for independent IL-2Rα and AUC = 0.973 for IL-2Rα combining with IP-10) (**Supplementary Figures 6A, B**).

### Combining IL-2Rα and IP-10 may improve the value of predicting the progression of symptomatic neurosyphilis

**Figure 4:**
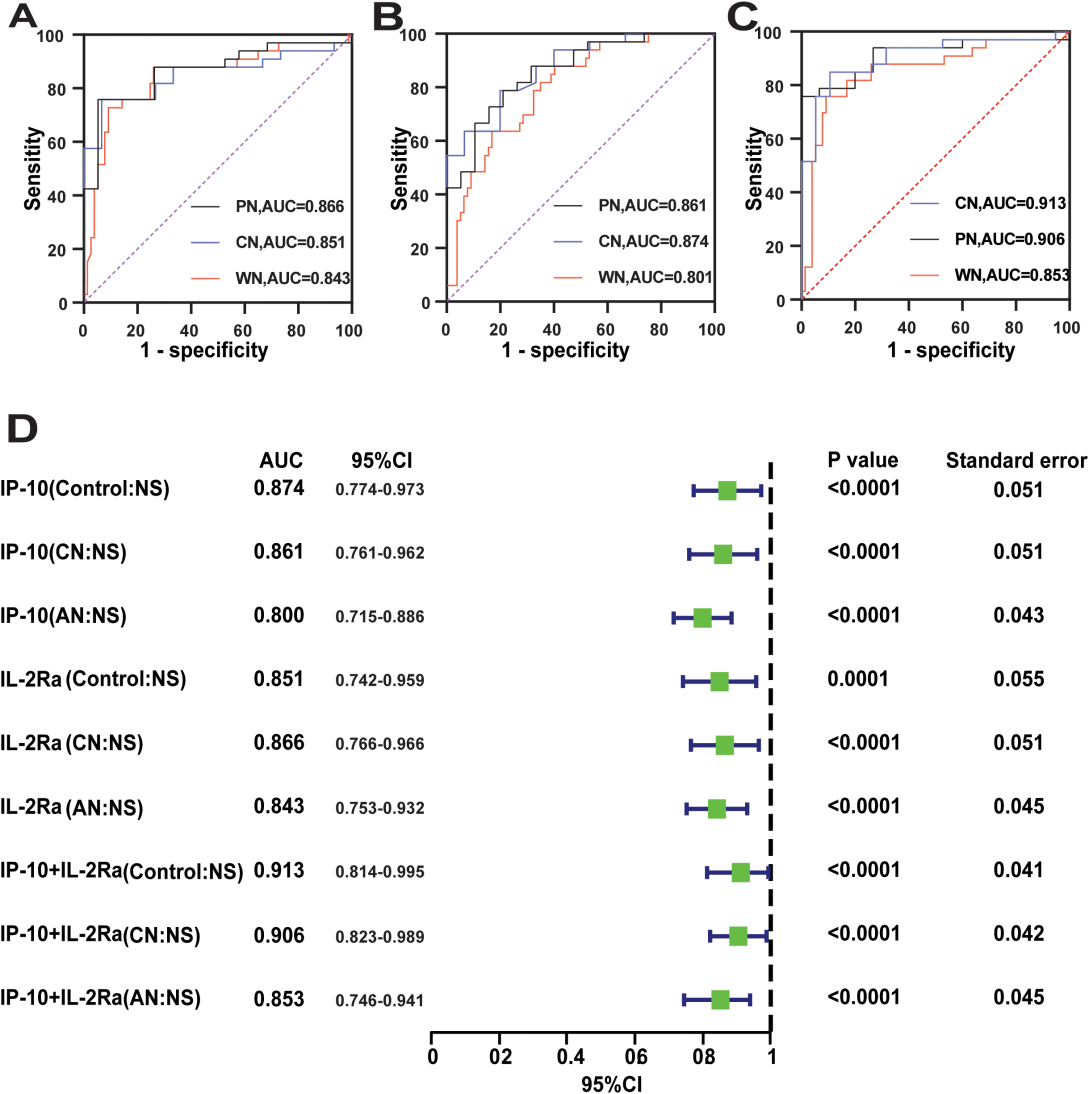
The ability of IL-2Ra and IP-10 to predict disease progression in neurosyphilis. **(A–C)** The ROC of IL-2Ra, IP-10 and IL-2Ra+IP-10, PN(CN: NS), CN(Control: SN), WN(AN: NS). **(D)** The forest plot showed the predictive effect of IL-2Ra, IP-10 and IL-2Ra+IP-10 for different immune response through ROC curve analysis.

The ROC curve analysis of the machine learning models suggested that combining IL-2Rα and IP-10 could improve performance. To verify this conclusion and further demonstrate the predictive effects of these two cytokines when comparing the CS and Control groups, we used traditional ROC curves to separately plot the predictive ROC curves of these cytokines alone and in combination for NS: Control, NS: CS, and NS: AN. It was a significant increase in AUC values compared to the ROC curves for IL-2Rα (AUC of 0.866, 0.851 and 0.843) (**Figures 4A, D**) and IP-10(0.874, 0.861 and 0.800) alone (**Figures 4B, D**). The results showed that combination of IL-2Rα and IP-10 displayed the highest AUC of 0.913, 0.906 and 0.853 (**Figures 4C, D**). These results suggested that IL-2Rα and IP-10 may be key cytokines in causing clinical symptoms of neurosyphilis, and their combination can effectively predict the disease progression of neurosyphilis.

### Age-Adjusted Multivariable Analysis and Age-stratified diagnostic performance

Given the significant age disparity between NS and AN patients (57.0 ± 2.3 vs. 38.3 ± 1.5 years; P < 0.001; Cohen’s r = 0.51; **Supplementary Table 1**), we performed multivariable logistic regression to adjust for age as a potential confounder. While IL-2Rα remained significantly associated with symptomatic neurosyphilis after age adjustment (standardized β = 0.903; OR = 1.05; 95% CI, 1.03–1.08; P < 0.001), age demonstrated a stronger independent effect (standardized β = 1.357; OR = 1.11; 95% CI, 1.05–1.16; P < 0.001) (**Supplementary Figure 7A**). The predictive model combining IL-2Rα and age achieved an AUC of 0.876, representing a 3.9% improvement over IL-2Rα alone (AUC = 0.843), indicating that IL-2Rα provides incremental value beyond age (**Supplementary Figure 7B**).

**Figure 5:**
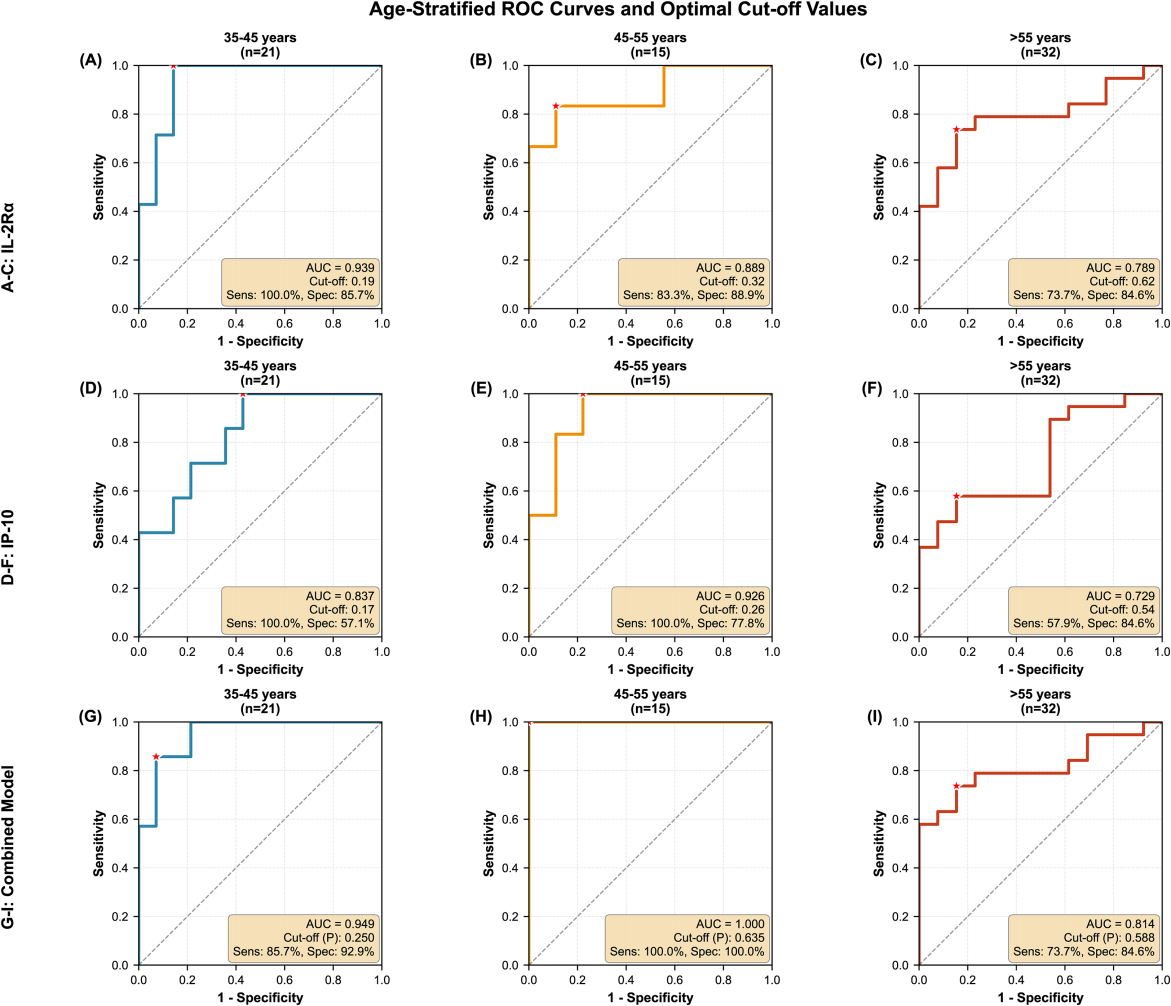
Age-stratified diagnostic performance of IL-2Rα, IP-10, and their combination for neurosyphilis prediction. Receiver operating characteristic (ROC) curves and optimal cut-off values were determined by logistic regression analysis with Youden index maximization for three age strata. **(A–C)** IL-2Rα alone; **(D–F)** IP-10 alone; **(G–I)** Combined model (IL-2Rα + IP-10). Columns represent age groups: 35–45 years **(A, D, G)**, 45–55 years **(B, E, H)**, and >55 years **(C, F, I)**. Red stars indicate optimal cut-off points determined by the maximum Youden index. The wheat-shaded boxes display the area under the curve (AUC), optimal cut-off values (probability for the combined model), sensitivity (Sens), and specificity (Spec) for each stratum. The diagonal dashed lines represent the reference line (AUC = 0.5). Sample sizes (n) are indicated in parentheses within each subplot title.

Recognizing that age represents both a demographic risk factor and a potential confounder of inflammatory biomarker levels, we performed stratified ROC analyses (**Figure 5** and **Supplementary Table 2**). IL-2Rα demonstrated robust diagnostic utility across all age strata, with AUC values ranging from 0.789 (95% CI: 0.712–0.866) in the >55 years group to 0.939 (95% CI: 0.885–0.993) in the 35–45 years group (**Figures 5A–C**, **Supplementary Table 2**). The optimal cut-off values for IL-2Rα exhibited age-dependent variation: 21.82 U/mL for 35–45 years (Sensitivity: 100.0%, Specificity: 85.7%); 25.48 U/mL for 45–55 years (Sensitivity: 83.3%, Specificity: 88.9%); and 24.54 U/mL for >55 years (Sensitivity: 73.7%, Specificity: 84.6%). Similarly, IP-10 showed stratum-specific performance across age groups (AUC: 0.729–0.926), with cut-offs ranging from 256.41 pg/mL in the 35–45 years group to 641.18 pg/mL in the 45–55 years group, and 597.64 pg/mL in the >55 years group (**Figures 5D–F**, **Supplementary Table 2**). Importantly, the combined model IL–2Rα+IP–10 achieved superior discrimination in all age groups (AUC: 0.814–1.000), with optimal probability cut-offs varying by age stratum: P ≥ 0.250 (35–45 years), P ≥ 0.635 (45–55 years), and P ≥ 0.588 (>55 years) (**Figures 5G–I**, **Supplementary Table 2**). Notably, the combined model achieved perfect discrimination in the 45–55 years cohort AUC = 1.000. These findings demonstrate that while absolute cytokine levels vary with age, both markers retain independent predictive value beyond age alone.

## Discussion

The nervous system may be invaded by Treponema pallidum even during early infection, and untimely or irrational treatment may progress to neurosyphilis, seriously affecting the patient’s quality of life (6, 30). The pathogenesis of neurosyphilis is still unclear, and the immune response is likely to be an important factor influencing the progression of neurosyphilis. Recently, it has been shown that IL-17A and interferon-γ are involved in the pro-inflammatory immune response in NS (31). IL-10, a potent anti-inflammatory cytokine, has been observed to be overproduced in advanced syphilis, and the overproduction of IL-10, which may promote persistent bacterial infections, plays an important role in the pathogenesis of NS and correlates with the progression of the disease (7, 32, 33). The diagnosis of neurosyphilis is mainly relied on clinical and laboratory parameters that is not enough sensitive and specific (34). Chemokines are expressed in both neuronal and glial cells of the CSF, the levels of various chemokines are elevated and associated with the severity or progression of several CNS inflammatory disorders (35–38). Previous studies have reported some identify laboratory tests with high sensitivity and specificity for diagnosis neurosyphilis. Many studies have demonstrated that the level of CXCL13 may be the potential biomarker for diagnosis or predicting the progression of neurosyphilis (2, 20, 34, 35). Wurong Li et al. reported that triggering receptor expressed on TREM2 levels elevated of CSF in neurosyphilis suggesting that TREM2 may be a valuable biomarker to quantity microglia activation in neurosyphilis and displayed the progression of neurosyphilis (21). However, there are few studies on the immune response in patients with neurosyphilis.

In this study, we explored the levels of CSF cytokines with markers of neuronal injury and explored the differences of these CSF cytokines between the NS and control, AN, CS groups, and found the diagnosis value of these cytokines. Three cytokines (RANTES, MIP-1b and IL-3) were a significant difference syphilis patients compared to the control group. The ROC curve(AUC = 0.869) combining these three cytokines indicates that have significant value for diagnosing syphilis patients. The cytokine panel distinguishing syphilis patients from non-syphilitic controls was enriched for chemokines involved in monocyte and T-cell recruitment (e.g., RANTES, MIP-1β), consistent with prior evidence of innate and adaptive immune engagement during *TP* infection (39). We used LASSO regression analysis, machine learning, and traditional ROC curve methods to further analyse the differences in cytokine expression among different syphilis patient groups. We found no significant differences in cytokine expression between the CS group and the AN group. However, multiple cytokines showed significantly different expressions between the CS group and the NS group, as well as between the AN group and the NS group, all of which were upregulated. This suggests a heightened inflammatory state that may contribute to neurological damage. We identified IL-2Rα as the primary marker distinguishing asymptomatic from symptomatic neurosyphilis, highlighting the critical role of T-cell activation in triggering neurological symptoms. The added value of IP-10 (a chemokine induced by IFN-γ) further indicates that Th1-polarized inflammation contributes to symptom manifestation. Together, these findings imply that clinical neurosyphilis may not merely reflect pathogen burden, but rather a maladaptive host immune response culminating in neural injury (40).

IL-2Rα is a glycoprotein belonging to the cytokine receptor family (41). IL-2Rα plays a key role in the immune system and is involved in the regulation of a variety of physiological processes, such as cell proliferation, activation of IL-2Rα promotes T cell activation and proliferation, which in turn is involved in the regulation of cellular immune responses. Meanwhile, IL-2Rα has been widely evaluated for drug discovery and biomarker studies (41, 42). A study has shown a causal relationship between two cytokines (IL-2Rα and IP-10) and heart failure (HF) (43). IP-10 is a chemokine secreted in response to IFN-γ and plays an important role in activating and regulating inflammatory and immune responses *in vivo*. Currently, IP-10 has shown potential application in COVID-19, tuberculosis, sepsis, Kawasaki disease, cancer and other diseases (44). Prior studies have reported the value of IP-10 in the detection and treatment of neurosyphilis (15, 34).

Our age-stratified analysis addresses a critical limitation in previous studies by establishing age-specific diagnostic thresholds for neurosyphilis prediction. While previous reports have suggested that inflammatory markers merely reflect age-related immune senescence, our data demonstrate that IL-2Rα maintains significant diagnostic utility across all adult age groups when appropriately standardized (16, 40, 45). Notably, optimal cut-off values exhibited an age-dependent pattern, peaking in the middle-aged cohort (25.48 U/mL for 45–55 years) compared to younger (21.82 U/mL for 35–45 years) and older adults (24.54 U/mL for >55 years). This non-linear trajectory likely reflects the complex interplay between physiological age-related immune activation and disease-specific T-cell responses, rather than a simple linear increase in baseline inflammatory tone. This age-specific calibration is essential for clinical translation, as applying a universal cut-off would result in unacceptable rates of misclassification in either younger (false positives) or older (false negatives) populations.

Furthermore, the consistent superiority of the combined IL-2Rα/IP-10 model across all age strata (AUC: 0.814–1.000; ΔAUC: 0.01–0.11 compared to IL-2Rα alone) suggests that these biomarkers capture distinct pathophysiological pathways—IL-2Rα reflecting T-cell activation and IP-10 indicating interferon-γ-mediated chemotaxis—that remain relevant regardless of patient age. Strikingly, the combined model achieved perfect discrimination in the 45–55 years cohort (AUC = 1.000, 100% sensitivity and specificity), highlighting the particular utility of dual-marker assessment in this high-risk demographic. Clinicians should therefore utilize these age-adjusted cut-off values (21.82 U/mL, 25.48 U/mL, and 24.54 U/mL for respective age groups) rather than dismissing elevated cytokine levels as “merely age-related” in older patients presenting with suspected neurosyphilis.

These findings highlight key cytokines linked to neuroinflammatory severity. Previous studies have reported some potential biomarkers such as CXCL13, TREM2 and IL-10 for the diagnosis and prediction of neurosyphilis (2, 20, 34). And this study is the first to find that IL-2Rα may influence disease progression in neurosyphilis and IL-2Rα combined with IP-10 value of predicting neurosyphilis can be further improved. This study further enriched the field of biomarker research in neurosyphilis. Additionally, we discovered for the first time that the three cytokines RANTES, MIP-1b, and IL-3 have significant value in the combined diagnosis of syphilis. This finding is of great importance for the diagnosis of both syphilis and neurosyphilis.

The application of machine learning in this study highlights its potential to uncover complex, non-linear relationships within high-dimensional biological data that traditional statistical methods might overlook. By integrating multiple cytokine levels, our models achieved superior diagnostic and predictive performance, demonstrating the advantage of a multi-marker approach over single biomarkers (46). However, the ‘black-box’ nature of some algorithms can limit interpretability (47), and model performance is highly dependent on the quality and size of the training dataset, which is a common limitation in medical AI applications (48). Regarding the clinical practicality of IL-2Rα and IP-10, both cytokines can be readily quantified in CSF using commercially available multiplex immunoassays (e.g., Luminex platform), a technology increasingly accessible in major clinical laboratories. As our study protocol specified, CSF should ideally be collected at the initial clinical presentation, prior to any treatment, to reflect the true inflammatory state (2, 13, 15, 43). While our study establishes the strong predictive value of these markers (with AUCs up to 0.913), defining precise, universally applicable cut-off values for clinical decision-making will require validation in larger, prospective, multi-centre cohorts. Nonetheless, our findings lay a crucial foundation for developing a clinically actionable algorithm to stratify neurosyphilis risk and guide early intervention.

However, there are some limitations to this study. First, the control sample size was small, which may lead to bias in the results. Second, this study only observed the expression levels of cytokines and did not delve into their specific roles in the pathogenesis of neurosyphilis. In addition, only specific cytokines were included in this study, and there may be other unobserved cytokines associated with neurosyphilis. While we provide age-stratified cut-offs, the sample sizes within individual age strata (particularly the 35–45 years group) were limited, potentially affecting the precision of threshold estimates. And we acknowledge that this cross-sectional study design precludes determination of the temporal dynamics of cytokine elevation relative to symptom onset. All CSF samples were collected at a single time point (initial hospital admission, prior to treatment), reflecting the inflammatory state at presentation rather than disease progression. Longitudinal studies with serial CSF sampling are warranted to establish whether IL-2Rα and IP-10 elevation precedes symptom development or correlates with treatment response. Future multi-centre validation studies with larger, age-balanced cohorts are needed to refine these cut-offs and confirm their generalizability across diverse ethnic populations with varying baseline inflammaging profiles.

## Conclusion

In conclusion, IL-2Rα and IP-10 represent promising predictive biomarkers for neurosyphilis progression, with their combined application achieving high diagnostic accuracy for distinguishing symptomatic from asymptomatic disease. Despite the age disparity between patient groups, multivariable analysis confirmed that IL-2Rα contributes independent prognostic information beyond chronological age, supporting its utility as a severity indicator and potential predictor of neurological deterioration. These findings establish a foundation for CSF cytokine-guided risk stratification in syphilis patients, although prospective validation in larger, age-matched cohorts is warranted to optimize clinical cut-offs and confirm generalizability across diverse populations.

## Data Availability

The original contributions presented in the study are included in the article/**Supplementary Material**. Further inquiries can be directed to the corresponding authors.

## Supplementary material

Supplementary Figure 1 The overall workflow of this study.

Supplementary Figure 2 Basic characteristics and clinical symptoms of subjects. **(A)** show the distribution of gender. **(B)** show the distribution of age. **(C)** show the discrepancy of time of viral infection between CS, AN and NS groups. **(D)** show the discrepancy of BMI between CS, AN and NS groups.

Supplementary Figure 3 Scatterplot showing 16 of the 48 cytokines with relatively high variability between groups.

Supplementary Figure 4 Traditional ROC curve validation of 3 screened cytokines. **(A)** Individual ROC curves for the three cytokines MIP-1b, IL-3 and RANTES. **(B)** ROC curve of MIP-1b in combination with IL-3. **(C)** ROC curve of RANTES combined with IL-3. **(D)** ROC curve of RANTES combined with MIP-1b.

Supplementary Figure 5 Comparison of cytokines among the CS group and Neurosyphilis (AN and NS) in CSF samples. **(A)** PCoA of cytokine among CS group and Neurosyphilis (AN and NS) in CSF samples. Each plot represents one sample. **(B)** Graph of difference bubbles illustrated the differential expression of 48 cytokines in Control and syphilis patient groups. **(C)** Lasso regression was further analysed for 8 differentially expressed cytokines among the Control and Syphilis groups. **(D)** Violin plot displaying the MIP-1a differentially expressed cytokines in **(C)**. *p < 0.05, **p < 0.01, ***p < 0.001. **(E)** Machine Learning Models Further demonstrate diagnostic and predictive capabilities of MIP-1a with significant differences. **(F)** Traditional ROC curves characterise the detection efficiency of MIP-1a.

Supplementary Figure 6 Comparison of cytokines among the AN and NS in CSF samples. **(A)** Machine Learning Models Further demonstrate predictive capabilities of IL-2Ra. **(B)** Machine Learning Models Further demonstrate predictive capabilities of IL-2Ra combining with IP-10. **(C)** The confusion matrix for IL-2Ra **(D)** The feathure Importance and permutation Importance of the random forest model for IL-2Ra. **(E)** The confusion matrix for IL-2Ra+IP-10. **(F)** The feathure Importance and permutation Importance of the random forest model for IL-2Ra+IP-10.

Supplementary Figure 7 Independent predictive value of IL-2Rα for symptomatic neurosyphilis and effect of age adjustment. **(A)** Multivariable logistic regression analysis of factors associated with progression from asymptomatic (AN) to symptomatic neurosyphilis (NS). **(B)** Receiver operating characteristic (ROC) curve comparison demonstrating the incremental diagnostic value of IL-2Rα beyond chronological age.
