# Supplementary material for: Cytokines in cerebrospinal fluid combined with machine learning improve the diagnostic accuracy and predict the progression of neurosyphilis
Source: Front Immunol. 2026 Apr 20;17:1677008. doi: 10.3389/fimmu.2026.1677008 (PMC13135949; doi:10.3389/fimmu.2026.1677008)
Supplement: Supplementary file 1 [file Table1.docx]

Table S1. Epidemiologic and clinical features of patients with syphilis in this study

| Characteristic | CS**(n=19)** | AN**(n=77)** | NS(n=33) | P value(χ2 test/fisher/one-way ANOVE test) |
| --- | --- | --- | --- | --- |
| Age (years), mean ± SD | 41.8 ± 3.0^a^ | 38.3 ± 1.5^a^ | 57.0 ± 2.3^b^ | *P=*0.001 |
| Age(years old), n (%) |  |  |  |  |
| <20 | 0 (0.0%)a | 2 (2.6%)a,b | 0 (0.0%)a | *P<*0.001 |
| 20-40 | 7 (36.8%)a | 45 (58.4%)b | 1 (3.0%)a |  |
| 40-60 | 11(57.9%)a | 24(31.2%)a | 22(66.7%)a |  |
| ≥60 | 1(0.52%)a | 6(0.78%)a | 10(30.3%)a |  |
| Sex，male | 7/19(36.8%) | 37/77(42.8%) | 22/33(66.7%) | *P=*0.081 |
| BMI(kg/m²), mean ± SD | 23.7 ± 0.7 | 23.3 ± 0.4 | 23.5 ± 0.6 | *P=*0.886 |
| Infected history (years) |  |  |  |  |
| < 1 | 4(21.0%) | 21(27.3%) | 9(27.3%) | *P=*0.0927 |
| 1-3 | 9(47.4%) | 36(46.8%) | 16(48.5%) |  |
| 3-5 | 3(15.8%) | 14(18.2%) | 4(12.1%) |  |
| ≥5 | 2(10.5%) | 6(7.79%) | 5(15.2%) |  |
| CSF WBC(cells/μL),mean ± SD | 1.9 ± 5.51 | 6.3 ± 2.7 | 8.7 ± 4.3 | *P=*0.457 |
| Cerebrospinal Fluid Protein (mg/dL) ,mean ± SD | 178.1 ± 45.9a | 73.6 ± 22.8a | 143.8 ± 35.9b | *P=*0.001 |
| Blood WBC(×10⁹/L),mean ± SD | 6.4±1.8 | 7.4±2.1 | 7.1±2.0 | *P=*0.570 |
| Co-existing condition | 9/19（ 47.4%） | 44/77 （57.1%） | 21/33（ 63.6%） | *P=*0.52 |
| High blood pressure | 4/19（ 21.1%） | 7/77（ 9.1%） | 8/33（ 24.2%） | *P=*0.072 |
| Diabetes | 0/19 （ 0.0%） | 4/77（ 5.2%） | 4/33（ 12.1%） | *P=*0.245 |
| Heart disease | 0/19 （0.0%） | 1/77（ 1.3%） | 2/33（ 6.1%） | *P=*0.214 |
| Hyperlipidaemia | 0/19（ 0.0%） | 3/77 （ 3.9%） | 2/33 （ 6.1%） | *P=*0.669 |
| Depression | 0/19 （0.0%） | 2/77（ 2.6%） | 0/33（ 0.0%） | *P=*0.504 |
| Cerebral Infarction | 0/19 ( 0.0%) | 2/77( 2.6%) | 4/33( 12.1%) | *P=*0.094 |
| HIV (Human Immunodeficiency Virus) | 1/19( 5.3%) | 1/77( 1.3%) | 0/33( 0.0%) | *P=*0.335 |
| HBV | 1/19( 5.3%) | 3/77 (3.9%) | 0/33( 0.0%) | *P=*0.477 |
| Other | 4/19( 21.1%) | 30/77( 39.0%) | 12/33( 36.4%) | *P<*0.001 |

Note. Data were represented as mean ± SD or n/N. a, b and a.b The same letter criterion indicates no statistically significant difference between groups, and different letters indicate a statistically significant difference, a.b indicates no statistical difference from both a and b.

****Table S2.**** Age-stratified diagnostic performance and optimal cut-off values for cerebrospinal fluid biomarkers in distinguishing symptomatic from asymptomatic neurosyphilis.

| **Age group** | **Biomarker** | **n**  **(AN/NS)** | **Cut-off value** | **AUC (95% CI)** | **Sensitivity**  **(%)** | **Specificity**  **(%)** | **Youden Index**  **(J)** |
| --- | --- | --- | --- | --- | --- | --- | --- |
| **35–45 years** | IL-2Rα (U/mL) | 21 | 21.82 | 0.939 (0.885–0.993) | 100.0 | 85.7 | 0.857 |
|  | IP-10 (pg/mL) | 21 | 256.41 | 0.837 (0.756–0.918) | 100.0 | 57.1 | 0.571 |
|  | Combined (Probability) | 21 | P ≥ 0.250 | 0.949 (0.901–0.997) | 85.7 | 92.9 | 0.786 |
| **45–55 years** | IL-2Rα (U/mL) | 15 | 25.48 | 0.889 (0.823–0.955) | 83.3 | 88.9 | 0.722 |
|  | IP-10 (pg/mL) | 15 | 641.18 | 0.926 (0.867–0.985) | 100.0 | 77.8 | 0.778 |
|  | Combined (Probability) | 15 | P ≥ 0.635 | 1.000 (1.000–1.000) | 100.0 | 100.0 | 1.000 |
| **>55 years** | IL-2Rα (U/mL) | 32 | 24.54 | 0.789 (0.712–0.866) | 73.7 | 84.6 | 0.584 |
|  | IP-10 (pg/mL) | 32 | 597.64 | 0.729 (0.643–0.815) | 57.9 | 84.6 | 0.425 |
|  | Combined (Probability) | 32 | P ≥ 0.588 | 0.814 (0.736–0.892) | 73.7 | 84.6 | 0.584 |
| **Overall** | IL-2Rα (U/mL) | 110 | 20.68 | 0.843 (0.786–0.900) | 87.9 | 81.8 | 0.697 |
|  | IP-10 (pg/mL) | 110 | 466.38 | 0.800 (0.740–0.860) | 81.8 | 76.6 | 0.584 |
|  | Combined (Probability) | 110 | P ≥ 0.374 | 0.853 (0.796–0.910) | 87.9 | 85.7 | 0.736 |

Notes: IL-2Rα concentrations are expressed in U/mL; IP-10 concentrations are expressed in pg/mL. Combined model cut - offs represent probability thresholds (range: 0–1). Optimal cut - off values were determined by maximizing the Youden index (J = Sensitivity + Specificity − 1) within each age stratum. 95% confidence intervals for AUC were calculated using the Delong method.
