# Supplementary figures and images for: Cytokines in cerebrospinal fluid combined with machine learning improve the diagnostic accuracy and predict the progression of neurosyphilis
Source: Front Immunol. 2026 Apr 20;17:1677008. doi: 10.3389/fimmu.2026.1677008 (PMC13135949; doi:10.3389/fimmu.2026.1677008)

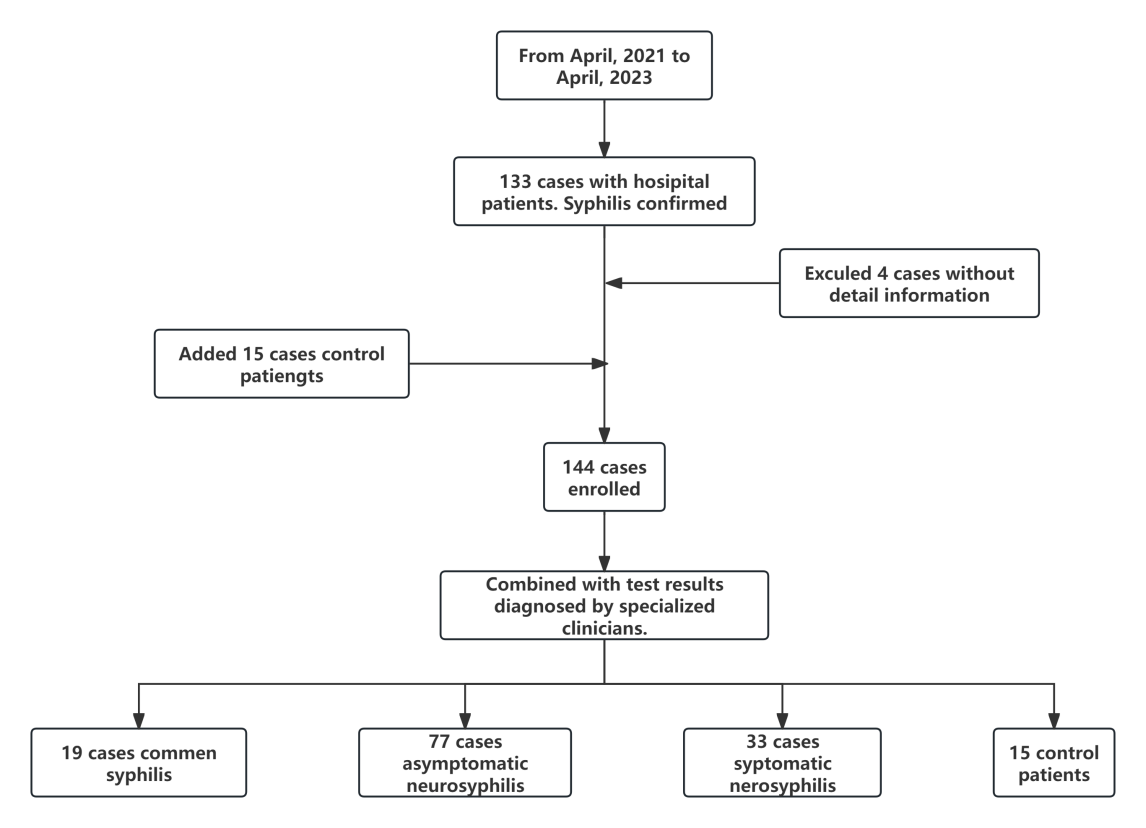

Supplement: Supplementary Figure 1 — The overall workflow of this study. [file Image1.tif]

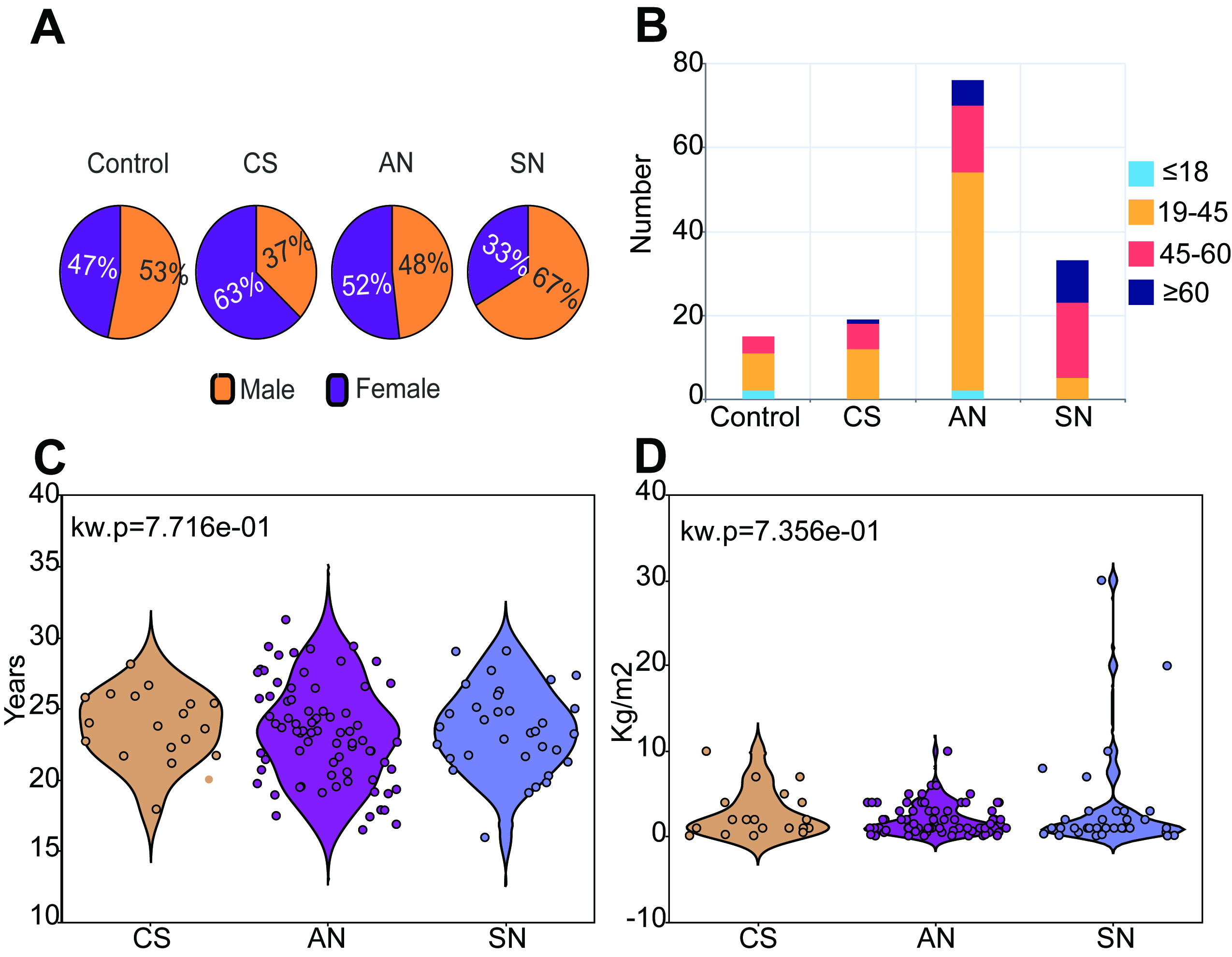

Supplement: Supplementary Figure 2 — Basic characteristics and clinical symptoms of subjects. (A) show the distribution of gender. (B) show the distribution of age. (C) show the discrepancy of time of viral infection between CS, AN and NS groups. (D) show the discrepancy of BMI between CS, AN and NS groups. [file Image2.tif]

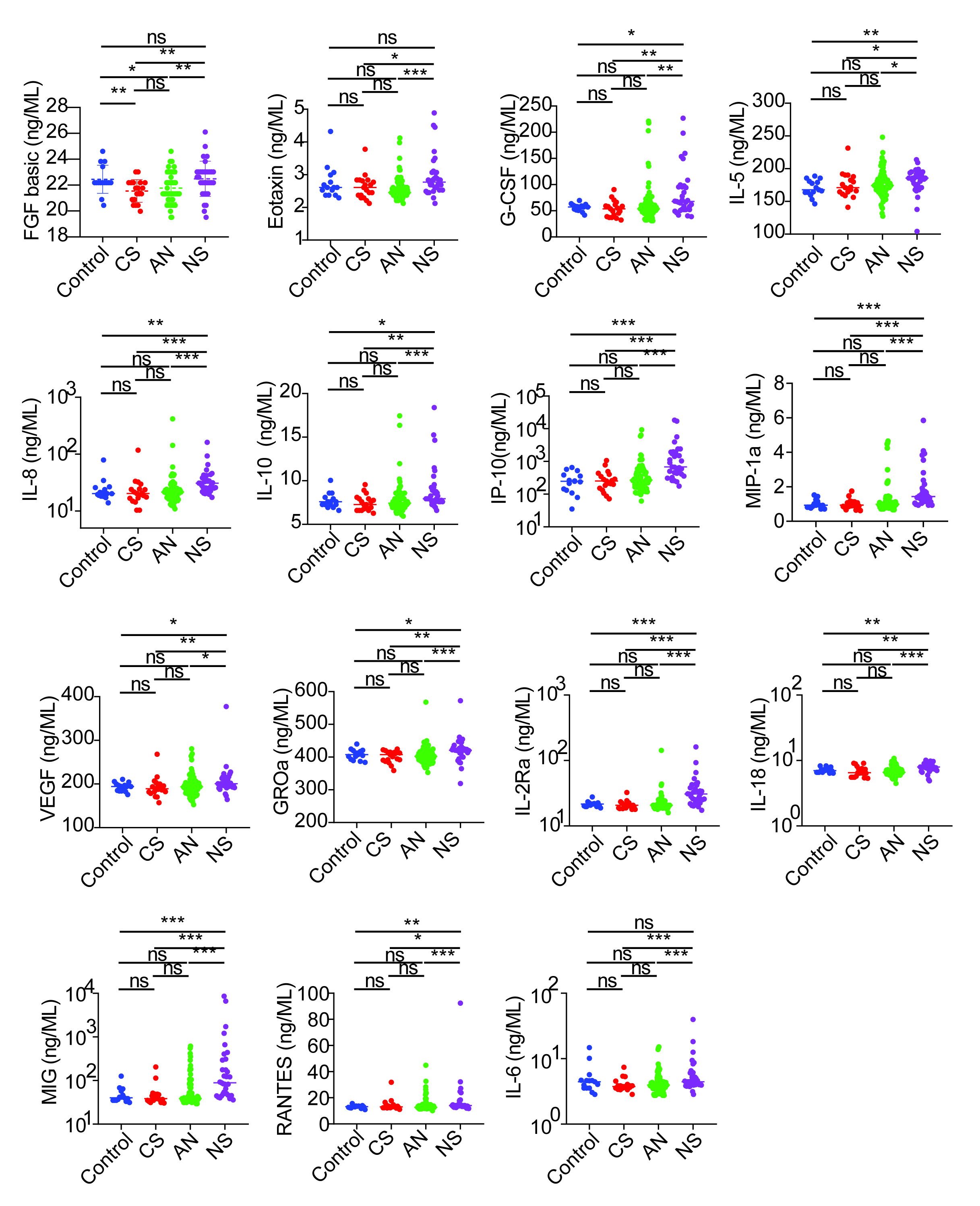

Supplement: Supplementary Figure 3 — Scatterplot showing 16 of the 48 cytokines with relatively high variability between groups. [file Image3.tif]

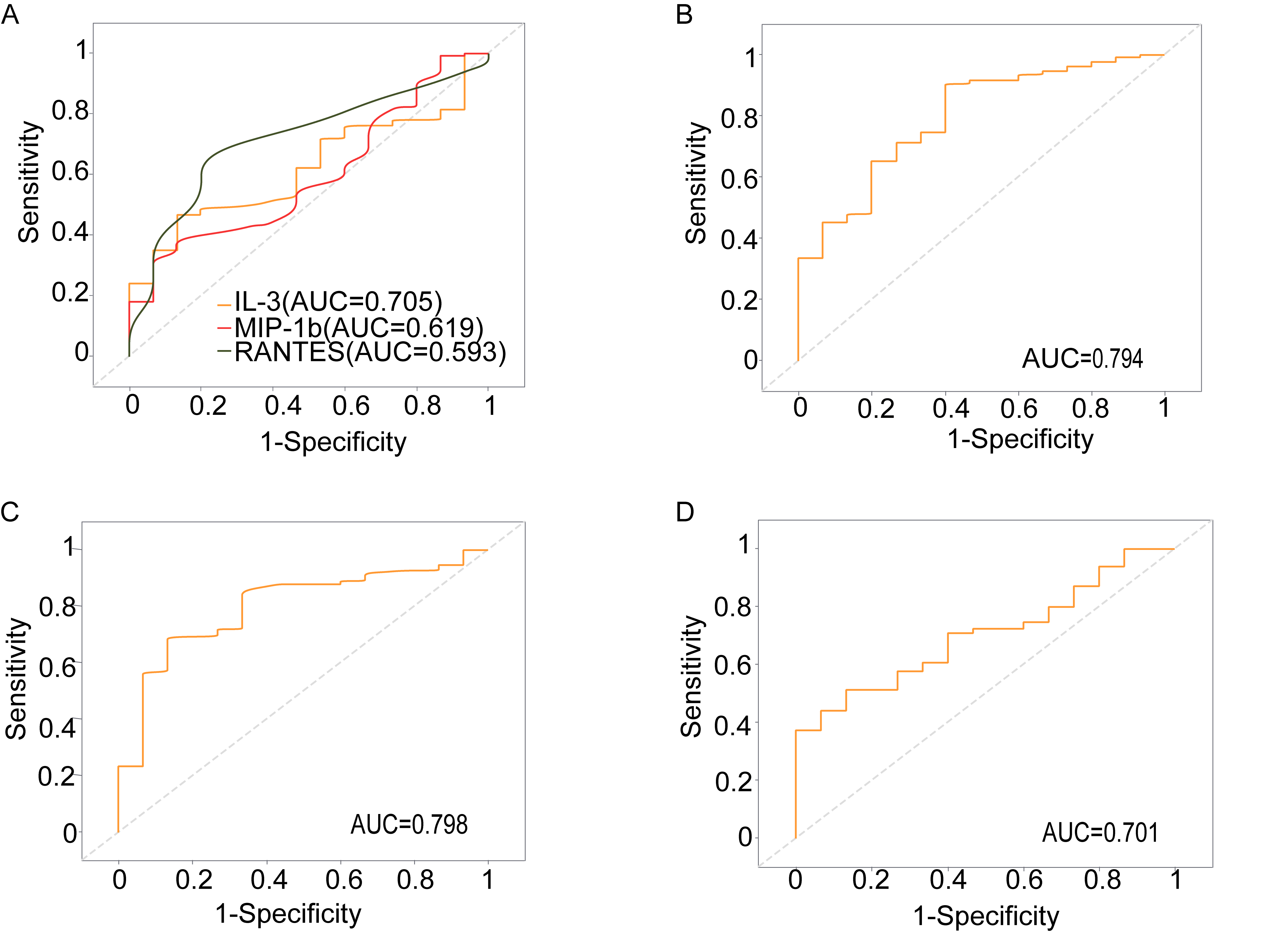

Supplement: Supplementary Figure 4 — Traditional ROC curve validation of 3 screened cytokines. (A) Individual ROC curves for the three cytokines MIP-1b, IL-3 and RANTES. (B) ROC curve of MIP-1b in combination with IL-3. (C) ROC curve of RANTES combined with IL-3. (D) ROC curve of RANTES combined with MIP-1b. [file Image4.tif]

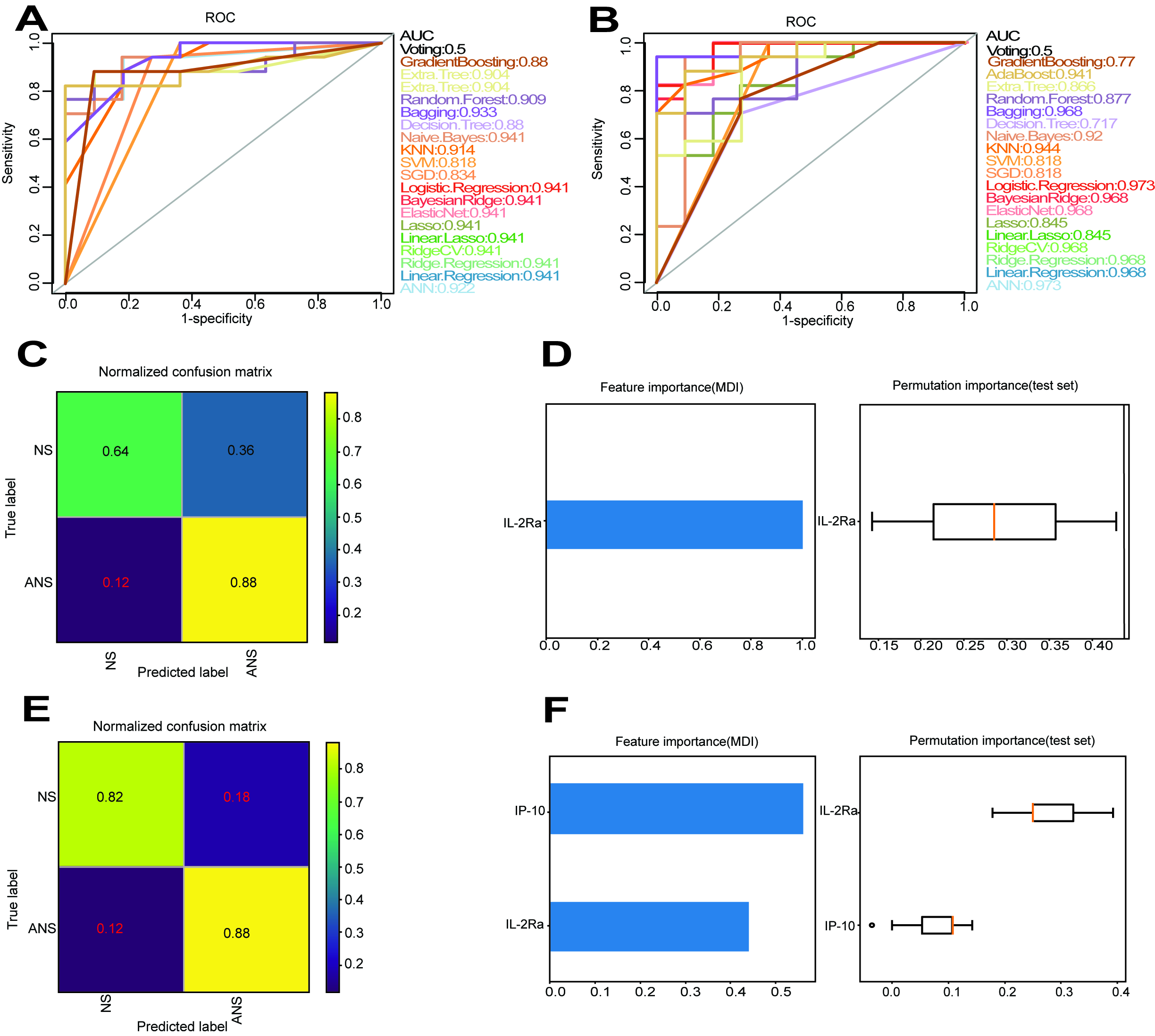

Supplement: Supplementary Figure 6 — Comparison of cytokines among the AN and NS in CSF samples. (A) Machine Learning Models Further demonstrate predictive capabilities of IL-2Ra. (B) Machine Learning Models Further demonstrate predictive capabilities of IL-2Ra combining with IP-10. (C) The confusion matrix for IL-2Ra (D) The feathure Importance and permutation Importance of the random forest model for IL-2Ra. (E) The confusion matrix for IL-2Ra+IP-10. (F) The feathure Importance and permutation Importance of the random forest model for IL-2Ra+IP-10. [file Image6.tif]

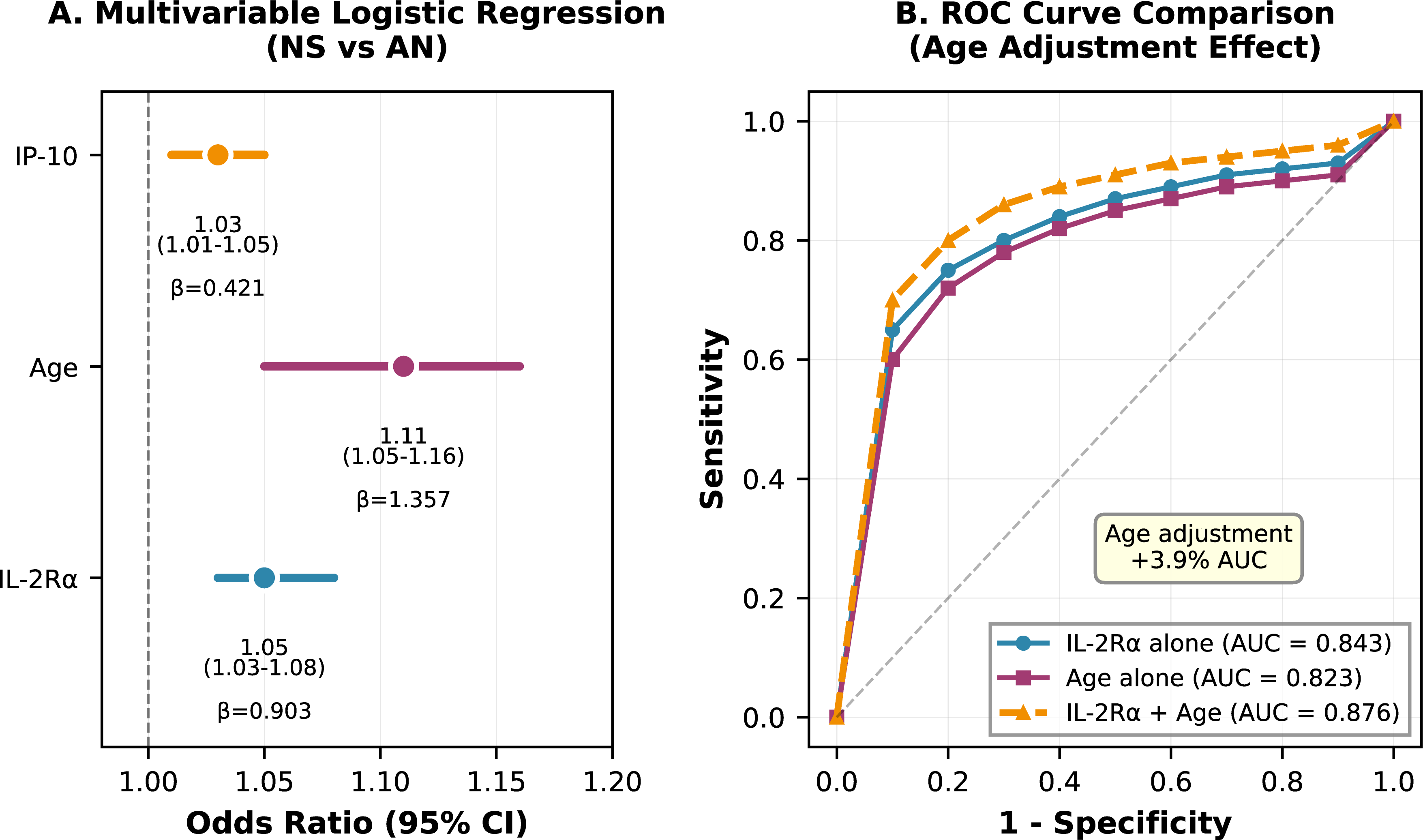

Supplement: Supplementary Figure 7 — Independent predictive value of IL-2Rα for symptomatic neurosyphilis and effect of age adjustment. (A) Multivariable logistic regression analysis of factors associated with progression from asymptomatic (AN) to symptomatic neurosyphilis (NS). (B) Receiver operating characteristic (ROC) curve comparison demonstrating the incremental diagnostic value of IL-2Rα beyond chronological age. [file Image7.tif]
